# Alcohol, the overlooked drug: clinical pharmacist perspectives on addressing alcohol in primary care

**DOI:** 10.1186/s13722-023-00378-x

**Published:** 2023-03-30

**Authors:** Mary Madden, Duncan Stewart, Thomas Mills, Jim McCambridge

**Affiliations:** 1 Department of Health Sciences, University of York, York, UK; 2 Centre for Primary Health and Social Care, School of Social Professions, London Metropolitan University, London, UK; 3 PHIRST South Bank, London South Bank University, London, UK

**Keywords:** Alcohol, Clinical pharmacy, Medication review, Brief intervention, Qualitative research

## Abstract

**Background:**

Attempts to routinely embed brief interventions in health systems have long been challenging, with healthcare professionals concerned about role adequacy, legitimacy, and support. This is the first study to explore clinical pharmacists’ experiences of discussing alcohol with patients in their new role in UK primary care, in developing a novel approach to brief intervention. It investigates their confidence with the subject of alcohol in routine practice and explores views on a new approach, integrating alcohol into the medication review as another drug directly linked to the patient’s health conditions and medicines, rather than a separated ‘healthy living’ issue. The study forms part of wider efforts to repurpose and reimagine the potential application of brief interventions and to rework their contents.

**Methods:**

Longitudinal qualitative study of 10 recruits to the new clinical pharmacist role in English primary care, involving three semi-structured interviews over approximately 16 months, supplemented by 10 one-off interviews with pharmacists already established in general practice.

**Results:**

When raised at all, enquiring about alcohol in medication reviews was described in terms of calculating dose and level of consumption, leading to crude advice to reduce drinking. The idea was that those who appeared dependent should be referred to specialist services, though few such referrals were recalled. Pharmacists acknowledged that they were not currently considering alcohol as a drug in their practice and were interested in learning more about this concept and the approach it entailed, particularly in relation to polypharmacy. Some recognised a linked need to enhance consultation skills.

**Conclusions:**

Alcohol complicates routine clinical care and adversely impacts patient outcomes, even for those drinking at seemingly unremarkable levels. Changing clinical practice on alcohol requires engaging with, and supportively challenging, routine practices and entrenched ideas of different kinds. Framing alcohol as a drug may help shift the focus from patients with alcohol problems to problems caused for patients by alcohol. This is less stigmatising and provides role legitimacy for pharmacists to address alcohol clinically in medication reviews, thus providing one element in the formation of a new prevention paradigm. This approach invites further innovations tailored to other healthcare professional roles.

**Supplementary Information:**

The online version contains supplementary material available at 10.1186/s13722-023-00378-x.

## Background

Pro-consumption social influences and the moralization of individual drinking make talking about alcohol challenging within contemporary health care systems that pay little attention to the social contexts of individual decision-making [1, 2]. Attempts to embed alcohol screening and brief interventions in health systems have been met with concerns from health care professionals about role adequacy, role legitimacy and role support [3–6]. Most research on alcohol in primary care is on General Practitioners (GPs) [7]. Thom and Tellez [8] identified addressing alcohol in GP consultations as a “difficult business” and Rapley and colleagues [4] concluded that not much had changed 20 years later. This study forms part of a programme attempting to establish a new prevention paradigm for alcohol within National Health Service (NHS) primary care [9, 10], which challenges the default framing of drinking as a decontextualized ‘lifestyle issue’ in medication reviews conducted by pharmacists [11, 12]. The Medicines and Alcohol Consultation (MAC) approach involves introducing alcohol as a drug [13], implicated in medication adherence, safety and effectiveness and thus a legitimate subject for discussion in medication reviews [14, 15]. Rather than asking pharmacists to take on an additional public health role [16], this integrates attention to alcohol within routine clinical practice and service delivery, in ways congruent with recognised good professional practice. The approach is the result of a co-produced intervention development process, with alcohol researchers working with people who drink alcohol and take medicines, community pharmacists and pharmacy academics [11, 17].

Investigation of routine practice and patient and pharmacist perspectives on the acceptability and suitability of the MAC approach in UK community pharmacy has shown the cursory nature of the alcohol consumption check as part of a ‘lifestyle’ section in routine medication reviews and wider uncertainties about the place of alcohol, and public health approaches more broadly [5, 18]. Pharmacists did not think they knew enough about the subject, did not feel it was their job to address alcohol issues beyond giving consumption advice, and felt left on their own to deal with any difficulties that might arise [5, 19].

A policy decision by NHS England to decommission key medication reviews from community pharmacy shifted our programme into primary care general practice, where a new clinical pharmacist (CP) role has been funded to deliver a new Structured Medication Review (SMR) service [20]. Implementation of the new patient-facing role and the SMR service was impacted by the COVID-19 pandemic; this, and how the new role fits into the emerging wider British primary care landscape has been reported on elsewhere [20–22]. Here, we focus on how pharmacists in this setting describe their alcohol practice, specifically their medication review practice, to explore the potential for, and receptivity to, the innovation involved in incorporation of the MAC approach. The SMR service specification highlights the risks to patient safety from alcohol interactions with medicines as something to be addressed within a thirty minute, clinically focused, holistic, medication review involving shared decision making [23]. The MAC approach is thus congruent with current policy directions and aspirations for practice. Understanding the existing practice context into which complex interventions are to be introduced is important because health professionals tailor implementation to their local practice needs [19, 24].

This qualitative study explores CPs’ reported experiences of their practice in discussing alcohol with patients in the general practice context, their confidence with the subject, and their views on introducing alcohol as a drug directly linked to the patient’s health conditions and medicines as part of routine medication review.

## Methods

The study received NHS Health Research Authority ethics and governance approval (REC reference 20/HRA/1482). The Covid-19 pandemic placed limitations on data collection in primary care, precluding direct observation of practice and calling for a pragmatic longitudinal approach as new CPs took time to settle into the patient-facing aspect of their role, delivering SMRs by telephone. Three in-depth semi-structured interviews were conducted with each of 10 newly appointed CPs between September 2020 and January 2022 (n. 30). After discussion with a pharmacy practice advisory group, researchers recruited CPs by telephone using a purposive sampling approach based on pharmacist workforce and SMR activity data.

In addition, a further 10 pharmacists already established in GP practices, were recruited using opportunistic sampling and snowballing techniques. This second group provided data on alcohol practice from pharmacists with longer experience of working in the general practice setting. Both groups were working in a pressured primary care landscape, experiencing many challenges including workforce shortages [22]. A leaflet describing the study and inviting pharmacists to contact the research team was distributed via national pharmacy organisations and on social media. The second group of longer established pharmacists were interviewed once between February and May 2021.

Interviews were conducted via video call by two experienced qualitative researchers (MM and TM) using a topic guide (see Additional file 1) which was informed by prior MAC intervention development studies and developed iteratively (interview total n. 40; pharmacist total n. 20). Audio-recordings were pseudonymised and professionally transcribed.

A modified framework method was used to organise and present data from transcripts [25]. Constructionist, thematic, comparative analysis identified common, recurring and conflicting perspectives, while noting the ways in which accounts were constructed [26]. The topic guide formed the initial framework for the first author when coding transcripts in NVivo 12. This produced a starting list of descriptive themes identifying alcohol practices and views on the MAC intervention, which was then subject to deeper analysis. Preliminary analysis of sample scripts, sub-themes through to the final analytic narrative were discussed with co-investigators. Our approach to reflexivity was grounded in the nature of this programme of studies, in which we had worked closely with patients, pharmacists and policy actors, and built the intervention content and research methods iteratively. Here we examined findings from our previous work, analytically reflecting on data here which might call these into question. These included how pharmacists pay cursory attention to alcohol presently, that clinical pharmacist expertise in medicines can provide role legitimacy to discuss alcohol, and that making these links salient in routine SMRs could go some way toward communicating the breadth and nature of the risks posed by alcohol consumption, thus challenging the view that alcohol only poses a problem for a stigmatised minority. Findings will inform further MAC development work in this setting [19].

All pharmacists were working with patients remotely, by telephone, with most of the new CPs yet to meet a patient face-to-face due to the Covid-19 pandemic. All of the 10 established GP practice pharmacists were prescribers and most were in, or taking on, senior and leadership roles. Five had previously worked in hospital pharmacy and three at commissioning level. One continued to work part-time in community pharmacy.

Three of the 10 new CPs were appointed at senior/lead pharmacist level, two of these had been qualified for 4 years and one for 30 years. One was provisionally registered, completing the necessary exams by the third interview. Tasks carried out by the new CPs changed over the three interviews as they became more established or moved post. Two of this cohort moved to a different location during the study; one moved twice. Eight had applied for their position from community pharmacy, one from hospital pharmacy and one from an existing GP practice pharmacist position. Two were prescribers. Some were working within one GP practice, while others split their time across practices. Most had pharmacist colleagues, but others were the first and sole pharmacist in their primary care setting. Further participant characteristics are in the Table 1. GP practice pharmacists are designated an X before their identifier number below to differentiate them from the new CPs.

**Table 1:** Participant characteristics (self-described)

Pharmacists	New clinical pharmacists	Existing general practice pharmacists
Age range	25–52	35–53
	mean 35.2	mean 41.8
	median 29.5	median 41.5
Gender identity		
Woman	7	8
Man	3	2
Ethnicity		
White British	8	7
British Pakistani	1	0
British Persian	1	0
British Indian	0	2
British Bangladeshi	0	1

## Results

### Routine alcohol practice in medication reviews

Of the more established GP practice cohort: three (X3, X6, X9) said they rarely or never raised alcohol in their medication reviews; five (X2, X4, X5, X7, X10) said they raised it sometimes, if they thought it relevant, or if it was flagged on the records; and two (X1, X8) said that it was on templates they had designed themselves and was therefore always or mostly raised (X1, X8). Six of the newly recruited CPs reported asking about alcohol in medicines reviews in response to alerts for missing information, or as part of completing a template series of healthy living questions at the end of a review (1, 3, 4, 5, 7, 9). One mostly doing care home SMRs with older people, said it was raised but rarely discussed (4). Another did not raise the subject in that setting because of the impression that it was not relevant (10). Over the course of the interviews, an additional three of this cohort reported starting to raise alcohol in reviews and clinics where they recognised it as an issue, including mood disorder reviews and warfarin, epilepsy and direct oral anticoagulant (DOAC) clinics (2, 6, 8). Most did so as a quick additional check.

In both cohorts, when the subject was raised, the practice described was very brief, reactive and formulaic:

I do ask sometimes as part of a review, but it’s, kind of, in a box ticking way … Rather than anything else really and secretly … hoping that I don’t unearth a huge problem that I have to deal with (8).

Most reported asking closed questions about consumption. They then gave generic advice, predominantly to drink at recommended levels and have drink free days, with limited, if any, tailoring to the patient’s own personal and health circumstances. Risk guidelines were treated more as universal targets rather than generic guidance to be tailored to circumstances, meaning that risks from alcohol connected to particular medicines and conditions were missed. Some of the pharmacists said they used, or referred patients to, a unit calculator and drink free day materials on the alcohol industry Drink Aware website [27] or the NHS ‘One You’ campaign website [28].

To guard against, or in response to negative reactions to questioning, some pharmacists said they told patients that they had to ask these questions and others stressed that they were asking for the patient’s wellbeing:

… I always blame, ‘them’, you know? ‘They’ve asked me to ask’ … I don’t actually say that, but … you sort of imply that you’re doing it because you have to (3).

This pharmacist and many of the others found leaving alcohol to the end of a review easier because that gave the chance to build up rapport:

… you’ve already shown that you’re friendly, and that they can tell you, and that you won’t shout at them (3).

One spoke about experimenting with their practice and working to develop a conversational style:

… when I first started doing SMRs, I found it difficult to ask those questions … But … I’ve … tried different ways of asking … So, it’s always in a very conversational tone ... it very much depends on how receptive the patient is to the discussion (4).

Another pharmacist was concerned that various health professionals repeating questions and giving information which people did not see as relevant to their own drinking was perceived as patronising:

… people just feel like you’re patronising them when you ask that question … I think … they know, often … that people should drink less, but it’s just another health person telling me about it … especially when they’re drinking probably a little bit over the guidance … it’s almost, like, it’s a bit of a triviality … ‘I drink … ten units more, but so what? … it’s not much, I’m not an alcoholic’ (9).

Two pharmacists, one from each cohort, reported not feeling the authority or status to offer alcohol advice to assertive patients. A GP pharmacist gave an example where an older man, “completely bit my head off” (X7), and a new CP in a practice predominantly catering to university students was uncomfortable when asked directly for advice by a young man taking anti-coagulants who was drinking heavily and blacking out:

… he [the patient] was asking me … ‘if the apixaban thins your blood and the alcohol does this …’ all these questions. And I just thought, this is a little bit beyond my capabilities … it was probably to do with the fact that he was a similar age. It just felt difficult to be … telling him, well, you shouldn’t be doing that, and … having a slightly older GP to tell him, it would have been … more … parental in a way. But equally, I know that in most surgeries you could be talking to much older people (8).

Most pharmacists recognised a lack of training about identifying the relevance of and managing alcohol in consultations. Derived from limited practice and their wider experience, they had different and sometimes contradictory views about drinking behaviours and of people’s willingness to be “honest” about their drinking. Most were sceptical about self-reports, with the minority framing this as a problem of recall rather than dishonesty, and some saying patients might be more honest with pharmacists than GPs. Many presumed that those drinking “too much” would not tell them this, or would say they were happy with the amount they drank, leaving a question mark over the point of asking and indicating the morally loaded nature of the interaction:

… I think it’s quite a difficult question to ask and get an honest answer about … the bottom line is that patients, as soon as you ask, know if they’re in the right or wrong. They can either honestly tell you that they don’t drink, or they can tell you that they don’t drink as much as they know that they do … or they do drink a lot and they admit they drink a lot … I think they already know what you're going to say … (6).

Practice wisdom was to double the amount stated:

… there’s a rule in general practice, from what my GPs have been saying … that … when you ask patients how much alcohol they have, always double it, because they won’t tell you straight … patients, they tell you what you want to know, what you want to hear (X4).

Some said they raised alcohol if they saw a clear direct impact on the medication under consideration, but there were blind spots:

… if it was a medication related to it, then I would bring it up at the time. If not, then I would bring it up at the end ... blood pressure medication … I would bring … up during the consultation. But … for example, a statin, aspirin, and … say ibuprofen, which I know is not a great drug but just for the sake of argument. There’s nothing that causes drowsiness there, there’s nothing that is going to cause a risk for alcohol, then I wouldn’t bring it up at the same time as those medications (X5).

This pharmacist addressed alcohol and drowsiness but not gastric irritation. Another said they focused more on immediate rather than long term effects, underestimating the impact of alcohol on medications and the conditions for which these are taken:

…. there’s not a lot of medication that would have a direct affect on alcohol … your mind is focussed on medication and the condition, both … you’re not thinking much about the long-term effects of alcohol (X4).

Pharmacists who did not drink, or drank little themselves, expressed particular difficulty in calibrating units. They also lacked confidence in talking about a subject in which they had little direct experience:

… that’s why I … feel not so well equipped to … discuss the thing, because … I barely drink at all … it’s so rare … So, I, kind of feel almost inexperienced in the area (8).

### Identifying and responding to heavy drinkers

The key focus of alcohol discussion, to the extent that there was any, was on identifying heavy drinking, but most pharmacists were not sure how to respond if they discovered this, other than to give the same routine advice. A pharmacist who did not usually raise alcohol gave an example of when it had come up unavoidably in a review because a test result indicated liver damage from heavy drinking:

... they’ll get … abnormal liver blood tests and … told from the receptionist over the phone … just reduce your alcohol intake and have your blood tests done again in a month. And they don’t come back for the repeat blood test. And that would be something that I would pick up on the medication review … I don’t think it’s something that I do very well (X9).

Most were unsure about what to do for patients they described as “alcoholic” or “addicted”, with some speaking in fatalistic terms about the possibility of intervention:

… I don’t think you can address it rationally because addiction is a very irrational thing. It’s an illness in itself … you learn that you can’t just explain and rationalise with people because addiction doesn’t work like that. It’s … a very difficult thing to address (10).

Pharmacists looked to pass these patients on but were unsure where. Most talked about making a referral back to a GP or on to an alcohol service. One pharmacist, who considered 20 UK units (160 g) per week a “really high amount” to be drinking, whereas others considered this less risky, noted that GPs “don’t feel confident enough” with the conversation themselves (7). One pharmacist gave patients the details of services and reported two patients making their own referral to alcohol services, “because they’re struggling with mental health, and we’ve discussed the connection with mental health and alcohol dependency” (4). No one else recalled an example of making a referral successfully, other than back to a GP. Some thought that specialist alcohol services were inaccessible or that patients would be reluctant to take up what was on offer:

… if they’ve only got an alcohol issue they don’t really like the thought of going to a drug and alcohol service (X10).

A more confident pharmacist gave an example of practice focused on encouragement with a patient on proton pump inhibitors and vitamin B (thiamine) who was drinking 70 UK units (560 g) a week and had cut down during lockdown:

… you’re saying you hate this [thiamine] medication, we’ll go through [them] all … you’re doing great, you’re cutting down … well if you cut down by this [further] amount, I’ll stop that and I’ll take that off your record (X1).

### Thoughts on the MAC

Further detail on alcohol practice was disclosed when pharmacists were asked about the novel MAC approach as they explained why it resonated with them, or why they thought it would not work. It took some time to explain the alcohol as a drug concept, in particular, focusing on the broader health implications of drinking while taking medications, rather than only in relation to heavy drinking and dependence. A few of the pharmacists said they already considered alcohol as a drug. However, the examples given were confined to particularly heavy drinkers. A dichotomized view of alcohol harm focusing only on severe dependence, rather than recognition of a continuum of harms, made it difficult to initially shift the focus away from patients having an alcohol problem towards problems caused for patients by alcohol. Once they began to consider alcohol as a drug impacting health more broadly, many were surprised at the current lack of attention to alcohol in medication reviews, including reflection on their own practice. Illustrative quotations on the appeal of the concept are in Table 2.

**Table 2:** Appeal to pharmacists of considering alcohol as another drug

It hadn’t occurred to me before, but it is a drug … I mean, the people on anti-depressants, people with anxiety those kind of things, it’s something to think about … whenever I speak to anyone with depression or anxiety, it’s probably worth asking about alcohol as well … I absolutely agree that it’s within our purview … I’ll confess to, I certainly don’t make it my priority in many conversations, but I do like the aspect of treating it like a drug … I like the idea of linking it to the various conditions, and linking it to medicine interactions … I’ve always found diet and exercise to be more important … I couldn’t give you a solid answer as to why alcohol was less important … I could certainly hear a case of it being made in diabetes, in depression, in any sort of opiate treatment. But then … to a lesser extent, in any sort of cardiovascular illness, any sort of renal illness, or like, obviously liver problems … I can certainly hear a case being made for it being a drug (3)
I understand what you’re saying because it is a drug … It’s a funny thing [not seeing it as such] … I don’t think it’s because it’s not important because I think it’s very important. I think the focus is always on review the drugs, review the medication. But that is not being perceived to be one, is it? So therefore it’ll only come into the conversation if it becomes a problem … it’s another drug that people don’t see as a drug … They see it as a … it’s just a glass of wine, or a gin every night (2)
… have we got specific resources for pharmacists on the effects of alcohol on the various conditions that we could learn from and use as part of a tool to discuss with patients? Because as much as we all have ideas of the impacts of alcohol … [it’s] not necessarily something that immediately comes to the forefront of my mind, unless it's somebody that's got significant resistant hypertension. So something like that, if it exists, would be great and I would love to know where that is and if not, why doesn’t it exist? … generally when I'm doing structured medication reviews, I … have … the healthy lifestyle bit on the end … So we do all the meds and the diseases and actually that’s a point is that we don't necessarily feed that in, it becomes like the add-on at the end to cover (4)
I think it gives you another lever in to talk about it and, I'll be honest, it's not something I'd considered picturing it on the end of the prescription as ethanol rather than a drink. That's a very interesting way of looking at it … often when people are talking about their medicines they're wanting to know what are the side effects, how is this going to harm me, is it going to or what are the benefits of it? So really if you get to ethanol you can say, well, the side effects or the toxicity can be this, this, this and this, benefit … it might make you feel nice at the time for a short period but … my ears have really pricked up … I think that actually would perhaps have more of an impact on the way somebody thinks about it than the worry of addiction, because I think addicts it’s a different mindset I guess. Whereas if you say … you’re taking this drug ethanol regularly, it does have toxic effects, it can increase the risk of cancer, of heart disease, of diabetes, of all the rest of it, dementia, list all these horrible things that can happen and just make sure that is very much in people’s minds. And that would hopefully encourage them to reduce their intake (10)
[That] definitely resonates with me … especially with a lot of the elderly … population who are on multiple drugs … it can affect their overall quality of life, because they’re not getting the best from their medications … I think we need to almost start targeting that area … for me personally, it is about the ones who don’t think that they’re drinking a lot, that’s the hardest one … ‘oh, I only … have a glass when I’m cooking, I maybe have a glass with my dinner, but I don’t really drink a lot’, I get to hear that quite a lot … I think alcohol’s going to be an issue … even in the more affluent areas … but the areas where you’ve got … more deprivation, I suspect …you’re going to struggle more … they would be the ones that you would need to look at, particularly where you’ve got younger families … (X8)
… it’s having indigestion every day, that could lead to problems with your oesophagus, it’s … your bowel habits not being right and … doesn’t have to even be as deep as your blood pressure medication. And most of all, what it does to your mental health and your sleep … Especially, if you’re looking at [it]… as another drug … we ask people if they buy any medicines, over the counter, antihistamines, painkillers, whatever. So, why shouldn’t we ask them what their intake of alcohol is? … you’ve given us a lot to think about actually then next time I ask somebody how much they drink (5)

For those with little experience of broaching the subject, there was a positive response to learning more about alcohol as another drug in the mix with medication, but the thought of any alcohol discussion still raised concerns about causing offence, adding further complexity to a review and apprehension about what to do when encountering heavy drinking. Illustrative quotations are in Table 3. Some began to talk about alcohol as a potential candidate for the development of their clinical role:

… it’s trying to find areas that aren’t already being done where we can then make ourselves indispensable (X3).

**Table 3:** Pharmacist concerns about discussing alcohol as another drug in the mix with medication

… it's a really good idea … I do agree that it's something that … affects medicine experts … Just obviously it's just a bit difficult conversation to have … it’s something that, unfortunately, just gets put down as, like, social history rather than something that could and does affect a lot of other things … it’s only when it’s, sort of, too late … you’re looking at liver tests or looking at issues that have arisen from alcohol … it’s only then tackled … [but] it's quite a personal thing and quite a difficult conversation to have (6)
I think if you had the right confidence and the right communication skills for the consultations … I think even discussing with you … I may overlook the fact that I’ve talked about their drugs, I’ve talked about alcohol in the essence of healthy living but not realising that it does affect actually your medication. The only time people probably ask that, ‘I’m taking antibiotics, does it affect with the alcohol’? … If you put it in as one of the drugs, I think it would really help, personally myself, I think I’d probably use this now in my next one … [but] how to handle the conversation? … (7)
That’s an interesting idea … It is weird, isn’t it? [not thought of as a drug] … it’s just it’s so culturally accepted … It’s a challenging one to intervene on … to do just normal medication reviews on that level is a challenge in itself … So adding something extra on is almost like an extra challenge, but they could work together [alcohol and medication] (9)
It’s a drug, at the end of the day, it should be classed as a drug … [but] another awkward conversation … (5)
I can’t 100 per cent say that I would’ve thought about it [as a drug] so that will probably be something different to me to try and incorporate. Probably mainly because I’d be a bit more worried about, well what … what if the patient does tell me that they drink 50 units a week, what do I do then? So I like to be fore-armed with all the information of what to do next … where to refer people to if we do find something that’s not ideal … it’s probably just the phrases that you use rather than going down a tick list … I think once you’ve got used to your own style of questions and things … I think it’s just developing my own way of broaching it, so it doesn’t sound like it’s just a tick box (X3)

A GP pharmacist with previous experience of promoting brief alcohol interventions said one-to-one approaches should be complemented by public policy interventions on alcohol availability and raising awareness of alcohol harms (X8). She recognised some of the impacts of alcohol as drug, including the negative impact on hypertension control, but with a focus on those drinking above 30 or 40 units per week (see Table 4). An emphasis on identifying those drinking very heavily and “educating” patients about recommended levels of drinking was common across all the interviews. A pharmacist confident that they already saw alcohol as a drug as part of their “holistic” approach, but again focused on particularly heavy drinkers, started to stretch the pharmacist’s potential role from an expert in medication to tackling the underlying causes of drinking:

So it is another drug but … also … can indicate if someone … needs help for other things such as … depression, anxiety, mental health … or is it being used as a crutch, do we need to look at counselling avenues? And not necessarily … tackle the alcohol dead on, but tackle the underlying issue … if someone’s drunk their [whole] life within sensible limits then all of a sudden have come under immense pressure at work … and seem to be drinking a bottle of wine every night, is there something we can do about the immense pressure, take that away before that [becomes] a chronic drinking problem? Is there … depression in there, is there something else that if we treat those things, in some respect we can actually solve the issues with the alcohol without … saying … you’re an alcoholic … categorising them … So potentially … we look … clinically … is there anything that we can do and then obviously explain that alcohol’s a depressant, it won’t make you feel any better, you know. Full of calories … (X1).

**Table 4:** Example of current practice on which to build

For me, it [alcohol] features quite highly, especially when … doing the DOAC reviews [direct oral anticoagulants], I found we hadn’t updated people’s alcohol status for aeons, and … if somebody’s … poorly controlled hypertensive and they’re drinking … in excess of 30, 40 units a week … alcohol we know has a negative direct correlation into hypertension control. So addressing that definitely helps rather than just giving them add on treatment and add on therapies over and over again … it has a massive impact on … not only a patient’s weight, but … overall longer term liver complications and also it can affect how certain drugs work in the system, because it’ll affect the pharmacokinetic properties of certain drugs, so it’s really, really important for me to address that … particularly in the elderly population, because they are on multiple drugs, lots of polypharmacy, and if they’re used to having a little tipple every night, well, by the end of the week, that’ll easily build up, so it’s again useful to educate them around having sort of alcohol free days and things like that, just to allow for things to recover. Most of them are probably like, ‘oh love, you know, I’m 85 now, love, am I really going to change?’ I don’t know, but you still have to have that conversation, and do it repeatedly to see if that will eventually sink in [X8]	

This practitioner thus appreciated the explicitly clinical role envisaged for delivery of the new SMS service during the interview. The appeal of extending their traditional information-giving role to advise that alcohol is a depressant drug with numerous and complex health implications was clear, but less clear was what this pharmacist thought they might be able do to help take away, “immense pressure at work” (X1).

### Reasons to not change alcohol practice

There was understandable resistance to the prospect of changing roles and practices. Some pharmacists relatively inexperienced in the GP setting were positive about the MAC approach but translated it back into their current practice terms, with alcohol as another thing to gain and give information about, rather than as intended, a means of pharmacists engaging patients in conversation about their own alcohol-related health concerns. A minority found reasons why the MAC approach was not possible or better than existing practice. A pharmacist who agreed that it made sense to include alcohol as another drug in medication reviews doubted that, even if thought about in these terms, practice would change because alcohol is not currently considered a priority in primary care and doing so would mean additional work:

… that’s not to say it shouldn’t be [a priority]. It’s just … I like the idea of treating it as a drug … I think there are so many priorities … if you’ve got ten priorities you don’t have any priorities, do you? (3).

Others said that GP practices would be reluctant to take on something perceived as additional work unless incentivised. A small minority in both cohorts continued to justify not including alcohol at all in medication reviews, fearing, from their own rather than the patients’ perspective, adding additional complexity within the constraints of their short consultations.

Similarly, although not currently speaking to patients about alcohol, some presumed that people, particularly in areas of high deprivation, would not want to discuss it, even though alcohol consumption and its associated risks in these areas may be high:

… I don’t think there’d be much of a return … I might be wrong, but knowing the patients now, that I do and knowing how deprived their area is, I don’t think they’d be too honest, from what I can see (X4).

## Discussion

This study showed that alcohol was still regarded as a ‘difficult business’ for primary care [4, 8], and not only for GPs. Many of the issues facing GPs in earlier studies were similar for these pharmacists, now more than 35 years on. The practical challenges of managing alcohol in consultations with people with multiple conditions in busy practices may have, if anything, become more challenging as demand has risen and GP workloads have become recognised at a high level as unsustainable [29]. The new pharmacist workforce, while potentially having more time to spend with patients, has traditionally played a limited patient-facing role and is entering clinical practice less experienced and skilled in consultations than GPs. In this sample, and in other studies, there was limited basic or post-registration training on alcohol [3, 5, 16, 30], and where this covered brief interventions (a term used by only one interviewee) it largely relied on older models of structured brief advice which have advanced little in decades [12, 31]. Alcohol often feels awkward for both practitioners and patients to talk about. The ways in which such a contentious subject is discussed in giving advice [32], or in other approaches [33], are likely to matter greatly.

Alcohol was largely a blind spot among the growing concerns about potential harms from polypharmacy and the burden for patients and health professionals of managing multiple treatments for long-term conditions [14, 34]. Recognition that alcohol itself is not pharmacologically inert challenged current medication review ideas and practices, which positioned drinking alcohol simply as a lifestyle issue separated from medication issues, and/or only as an issue relevant to those drinking very heavily. Pharmacists mostly described a paternalistic, one-way, style of alcohol risk communication delivered as part of the administration of routine tasks. This aimed at filling a presumed knowledge deficit about recommended guidance and the need for adherence to it. A reliance on repetition to “educate” people was at some remove from the medication review consultation policy discourse of patient-centred, shared decision-making [19].

The focus across the board was on identifying heavy drinkers, but pharmacists were at best uncertain how to support these people if they were found and were fatalistic about the prospect of making a difference once there was a “problem”. Stigmatizing ideas were widespread; perceptions of the drinking of others deemed problematic served to disguise that harms may result from alcohol consumption at much lower levels when medications are also being used [14, 35–39]. Such stigmatizing perceptions may thus play an important role in inhibiting pharmacists to develop their approach to addressing alcohol unless addressed. In terms of prevention, some pharmacists were concerned that little was being done and they were reacting too late, but overall the locus of attention remained on finding dependence, recognised only when severe, rather than any nuanced understanding of the risks associated with this behaviour, including in relation to medication [4].

The novel MAC intervention approach [11] went some way towards shifting the focus of these interviewees from patients having an alcohol problem to problems caused for patients by alcohol. Framing alcohol as a drug in this way is less stigmatising and provides role legitimacy for pharmacists to address alcohol clinically in medication reviews, thus providing one element in the formation of a new prevention paradigm for this profession [9]. The approach departs from regarding alcohol as a standalone prevention target decontextualised from clinical care, patient presenting needs and organisational constraints. Rather, it seeks to locate alcohol discussions at the nexus of patient agendas and clinical care, integrating attention to alcohol within the consultation as a necessary element of good pharmaceutical practice. This implies little attention to alcohol for some people and a lot more for others. As this approach is developed further, we anticipate that innovations in practice will result from practitioners learning from their patients and sharing this knowledge with researchers [40, 41].

Pharmacists were aware of their lack of training and that their usual practice was rule-based rather than evidence informed. Their discretion on whether and how to raise alcohol in medication reviews relied on their own experience, their concerns about how people might react, and assumptions about who needed alcohol-related information. This led to some unhelpful ideas, including people in relatively poorer economic areas perceived to be drinking more, and therefore in need of intervention, but less likely to engage or be “honest”, so it was therefore not really worth asking. There were presumptions also that alcohol was not an issue for older adults although this group are particularly vulnerable to adverse effects from concurrent alcohol and medication use [42]. Such ideas need to be engaged with, and challenged, in ways which support practitioners to focus with greater skill on the clinical issues they encounter, in order to optimise the contribution to population health made by health service delivery.

The study provides a rigorous, in-depth, qualitative investigation of pharmacists’ views on their patient-facing alcohol practice from a sample including both those new to, and already experienced in, working in the GP practice setting. Remote working during the COVID-19 pandemic placed limitations on pharmacists’ capacity for patient-facing work, and on data collection in primary care. Comparison with observation of actual rather than reported alcohol practice, and the perspectives of patients are needed to further ground the findings in the empirical realities of routine practice.

## Conclusions

Framing alcohol as a drug provoked pharmacist interest and provided role legitimacy to address alcohol clinically in medication reviews. Most pharmacists were struck by its omission as a drug and quickly began to see ways in which it was relevant to medicines and conditions, rather than something to record separately after a review as part of a healthy living exercise. Changing alcohol practice requires significant enhancement of consultation skills to facilitate a more explicitly person-centred approach. Practice development and time with patients for exploratory consultations matter, and better outcomes remain possible given high-level strategic support to provide these services. This developing approach provides an exemplar for this profession in the formation of a new prevention paradigm for alcohol. Other approaches, tailored to professional roles, values and training, and appropriate for people who may derive benefit, and the specific contexts in which such discussions unfold, are encouraged for both healthcare and other settings.

## Supplementary Information

**Additional file 1.** Interview Topic Guide.

## Data Availability

The data that support the findings of this study are not publicly available because they contain information that could compromise the privacy of research participants.

## Abbreviations

CP: Clinical pharmacist
GP: General practitioner
NHS: National Health Service
SMR: Structured medication review
MAC: Medicines and alcohol consultation
DOAC: Direct oral anticoagulant
