# Supplementary material for: Alcohol, the overlooked drug: clinical pharmacist perspectives on addressing alcohol in primary care
Source: Addict Sci Clin Pract. 2023 Mar 30;18:22. doi: 10.1186/s13722-023-00378-x (PMC10062268; doi:10.1186/s13722-023-00378-x)
Supplement: Supplementary file 1 — Additional file 1. Interview Topic Guide. [file 13722_2023_378_MOESM1_ESM.docx]

Topic guide (this paper reports findings on alcohol)

- Details of PCN role – job title, when took up current post (or first worked in general practice setting), overview of current roles, brief career history
- PCN experience – views and experiences of PCN, relationship with colleagues during transition to general practice setting, local arrangements for integrating, managing and supervising clinical pharmacists, PCN level of maturity, pros and cons of working within particular operational model, any role in the integration of new clinical pharmacists, autonomy to shape PCN direction
- Patient facing practice – current and past, what “person-centredness” means in own practice
- Training for person-centred consultation practice – experience and suitability of training to become a person-centred practitioner (incl. most valued and gaps), experience of current or prior CPPE pathway, views on current PCPEP via direct experience and supervising others
- Medication reviews – understanding of the new Structured Medication Review (SMR), local implementation of SMRs, personal approach to medication reviews, alcohol within medication reviews
- Alcohol
  - experience talking about alcohol with patients in GP setting; examples of when found challenging or when went particularly well, whether perceived as a particularly sensitive topic, views on role legitimacy
  - views on MAC approach - including alcohol in SMRs as another drug impacting on medicines and conditions for people drinking twice a week or more - not targeted only at heavy drinkers, fit in relation to existing screening or intervention practices, reflection on own drinking.
